# Low-grade central fibroblastic osteosarcoma may be differentiated from its mimicker desmoplastic fibroma by genetic analysis

**DOI:** 10.1186/s13569-018-0104-z

**Published:** 2018-08-23

**Authors:** Wangzhao Song, Eva van den Berg, Thomas C. Kwee, Paul C. Jutte, Anne-Marie Cleton-Jansen, Judith V. M. G. Bovée, Albert J. Suurmeijer

**Affiliations:** 1 Department of Pathology and Medical Biology, University Medical Center Groningen, University of Groningen, P.O. Box 30.001, 9700 RB Groningen, The Netherlands; 2 Department of Genetics, University Medical Center Groningen, University of Groningen, P.O. Box 30.001, 9700 RB Groningen, The Netherlands; 3 Department of Radiology, Nuclear Medicine and Molecular Imaging, University Medical Center Groningen, University of Groningen, P.O. Box 30.001, 9700 RB Groningen, The Netherlands; 4 Department of Orthopedic Surgery, University Medical Center Groningen, University of Groningen, P.O. Box 30.001, 9700 RB Groningen, The Netherlands; 5 Department of Pathology, Leiden University Medical Center, Leiden, The Netherlands

**Keywords:** Bone sarcoma, Desmoplastic fibroma, Low-grade osteosarcoma, CDK4, RB1

## Abstract

**Background:**

We studied two cases of rare fibrous bone tumors, namely desmoplastic fibroma (DF) and low-grade central osteosarcoma (LGCOS) resembling desmoplastic fibroma (DF-like LGCOS). As the clinical presentation, imaging features and histopathology of DF and DF-like LGOS show much overlap, the objective of this study was to investigate the value of cytogenetic analysis, molecular pathology and immunohistochemistry in discrimination of these two mimickers.

**Case presentation:**

A mutation in *CTNNB* (S45F) and nuclear beta-catenin immunostaining were observed in DF. DF-LGCOS had amplification of *CDK4* and showed strong nuclear expression of CDK4 by IHC. Moreover, the karyotype of DF-LGCOS showed an interstitial heterozygous deletion of the long arm of chromosome 13 (q12q32), associated with loss of the *RB1* tumor suppressor gene.

**Conclusions:**

Karyotyping and molecular genetic analysis may contribute to a conclusive diagnosis.

## Background

The histopathological diagnosis of bone tumors is usually rather straightforward, since the most common bone tumors show differentiation along osteoblastic or chondroblastic lines, and form bone matrix or cartilage, which usually can be easily detected in routinely stained tissue sections.

In the past decades, advances in the field of immunohistochemistry (IHC) and molecular pathology have allowed a precise diagnosis in difficult cases. Examples are IHC for *SATB2* to confirm a tentative diagnosis of osteosarcoma, molecular DNA analysis for nonrandom gene translocations to differentiate Ewing sarcoma from other round cell sarcomas, and detection of *H3F3A* mutations to accurately diagnose giant cell tumor of bone.

However, for fibrous tumors of bone the incremental value of IHC and DNA methods over standard basic histology is rather limited. This category of fibrous tumors of bone includes the desmoplastic fibroma (DF)—a rare, locally aggressive tumor—and fibrosarcoma—a tumor once considered to be very common, but currently a diagnosis of exclusion, that one is only allowed to make after having ruled out other spindle cell tumors, e.g. low grade myofibroblastic sarcoma, myoepithelial tumors, follicular dendritic cell tumors, synovial sarcoma, and, last but not least, a rare variant of low-grade central osteosarcoma (LGCOS) resembling desmoplastic fibroma (DF-like LGCOS).

By co-incidence, two patients with these rare bone tumors (DF and DF-LGCOS) were treated in our sarcoma center in the same week. In addition to IHC, we decided to apply classic cytogenetics and next generation sequencing (NGS), which proved to be very helpful in discriminating these two morphologic mimickers.

### Case presentation

**Fig. 1:**
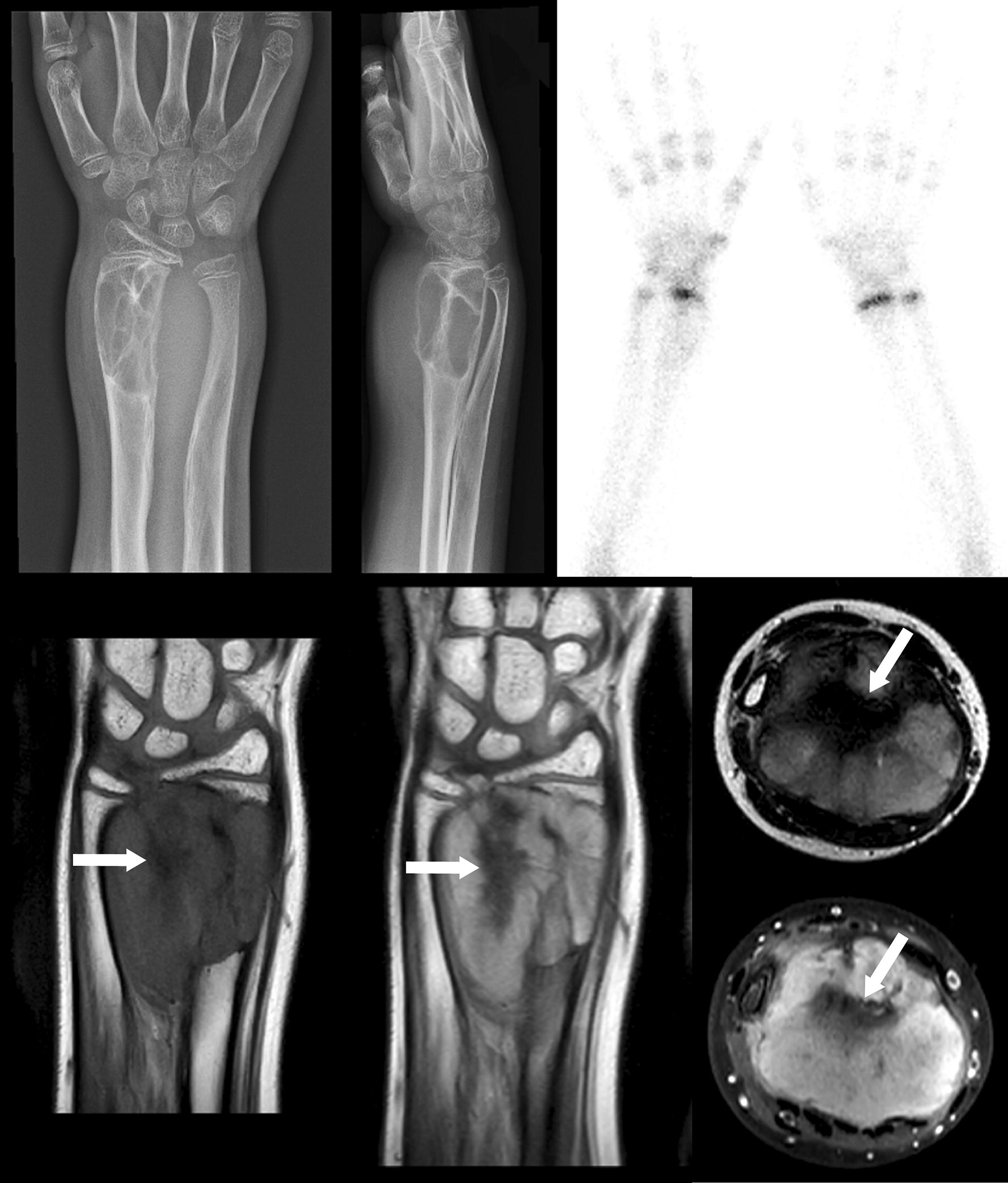
Conventional AP and lateral radiographs (top left and top middle) show an expansive bubbly lytic bone lesion in the diaphysis-metaphysis of the right distal radius with a narrow zone of transition, nonsclerotic margins, cortical thinning and destruction, and an accompanying large soft-tissue mass which appears to compress the distal ulna with bowing of the latter. Bone scintigraphy (top right) shows no increased uptake at the location of the lesion. MRI with coronal T1-weighted (bottom left) and gadolinium-enhanced T1-weighted (bottom middle) images, and axial T2-weighted and gadolinium-enhanced fat-suppressed T1-weighted images (bottom right) are in keeping with the conventional radiographic findings, and also demonstrate no signs of invasion in surrounding muscles or ulna. Remarkably, in the center of the lesion there is low signal on all sequences (arrows), most strikingly on the T2-weighted sequence. Because the combined imaging features suggest a slow-growing (most likely benign) process with fibrotic components, the differential diagnostic considerations include desmoplastic fibroma, and (less likely) giant-cell tumor or fibrous dysplasia

Case 1: a 10-year-old girl, with a history of distal radius fracture 3 years earlier, presented with a firm, nontender swelling in the same right distal forearm. Her wrist function was unimpaired. As shown in Fig. 1, X-ray examination revealed a large lobulated, compartmentalized, osteolytic, expansive tumor mass in the metadiaphysis of the distal radius. On MRI, the tumor measured 35 × 46 × 47 mm and had a well-defined boundary, but no sclerotic margin. Starting from the distal radius, there was cortical destruction, an extensive soft tissue component, and impression and bowing of the distal ulna. There were no imaging signs of invasive growth, necrosis or fluid-liquid mirrors. Bone scintigraphy did not show increased uptake at the location of the lesion. These imaging features were consistent with a destructive tumor that originated from the distal radius, grew slowly, and then broke through the cortex of the radius into the adjacent soft tissue. The tumor was excised intralesionally. Grossly, the largest tumor fragment measured 6 × 5 × 3 cm. On cut surface the tumor tissue was pale and fibrous.

**Fig. 2:**
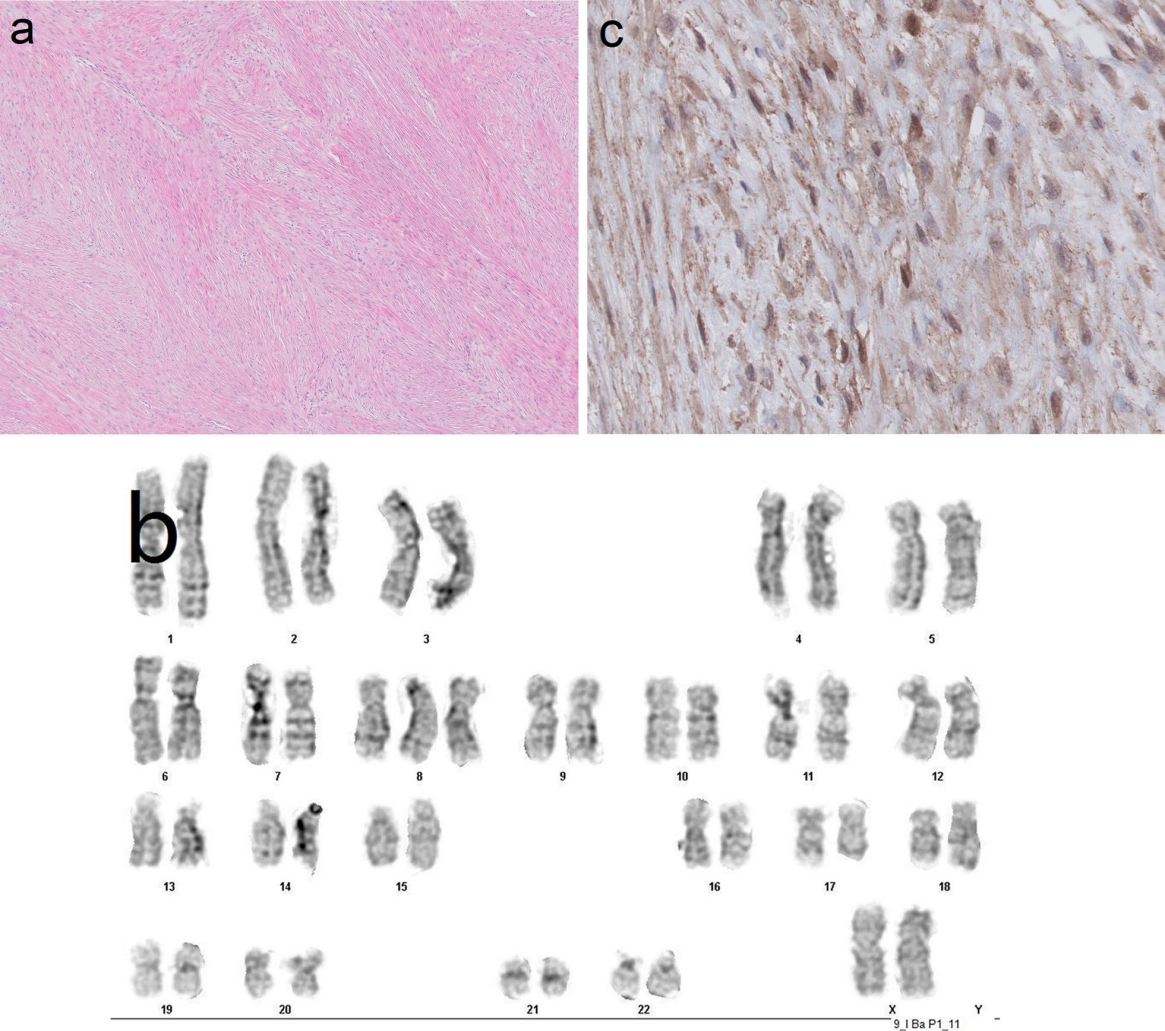
Histology, karyotype and beta-catenin nuclear expression in DF. **a** Histology showing a fibroblastic tumor with little or no nuclear atypia or mitotic activity (H&E, original magnification × 200). As such, the histology of DF resembles that DF-LGCOS shown in Fig. 4a. **b** DF karyotype: 47,XX,+8[2]/46,XX[18]. **c** IHC expression of beta-catenin in several tumor cell nuclei of DF (original magnification × 400)

Tumor histology was reminiscent of desmoid fibromatosis and consistent with desmoplastic fibroma, as it showed a lesion composed of bundles of moderately cellular, collagenous tumor tissue with fibroblastic spindle cells with oval, monomorphic nuclei with bland, finely granular chromatin, small nucleoli and ample cytoplasm. Mitoses were not found (Fig. 2a).

Cytogenetic analysis revealed a normal female karyotype in 18 cells, with trisomy 8 detected in 2 cells (Fig. 2b).

The cancer hotspot NGS analysis revealed a CTNNB1 hotspot class 5 pathogenic variant in exon 3: p.Ser45Phe and, using IHC, the fibroblastic tumor cells showed more than focal nuclear staining for beta-catenin (Fig. 2c), in support of a diagnosis of desmoplastic fibroma.

**Fig. 3:**
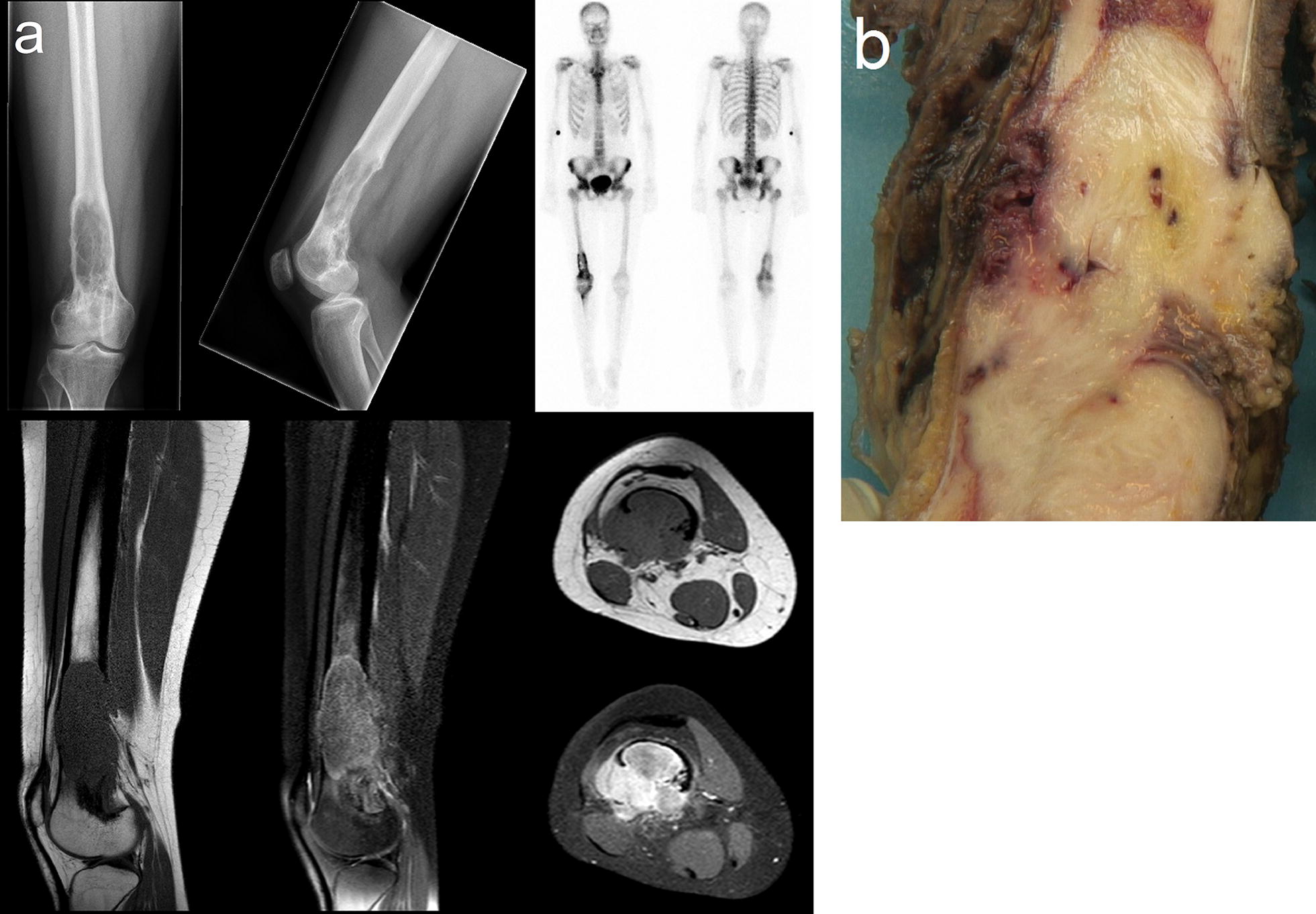
Conventional AP, lateral radiographs and gross morphology of DF-LGCOS. **a** Conventional AP, lateral radiographs (top left and top middle) show an expansive osteolytic lesion in the diaphysis-metaphysis of the right distal femur with an ill-defined border and cortical destruction. Bone scintigraphy (top right) demonstrates increased uptake at the location of the lesion, but no suspicious uptake elsewhere. MRI with sagittal T1-weighted (bottom left) and fat-suppressed proton density-weighted (bottom middle) images, and axial T1-weighted and gadolinium-enhanced fat-suppressed T1-weighted images (bottom right) show the T1 hypointense, T2 hyperintense, and vividly enhancing lesion in the right distal femur as a large soft-tissue mass with cortical breakthrough and extra-osseous expansion. The combined imaging features are highly suggestive of an aggressive malignant lesion, with osteosarcoma, Ewing sarcoma, and chondrosarcoma being the main differential diagnostic considerations. **b** Gross specimen of DF-LGCOS, showing a white, fibrous tumor of the distal femur with cortical breakthrough and invasion of soft tissue. As such, the gross appearance of DF-LGCOS resembles DF

Case 2: a 24-year-old woman presented with progressive pain in the right hip region that had existed for 1 year. X-ray images showed an osteolytic tumor in the metadiaphysis of the right distal femur with cortical bone destruction on the dorsolateral side. The central part of the tumor had no matrix calcification. On MRI, the tumor destroyed the cortex and extended to the surrounding soft tissues. There was strong tumor enhancement after administration of intravenous gadolinium (Fig. 3a). A resection of the right distal femur was performed. The tumor in the distal femur measured 12 × 4 cm. On cut surface the tumor was pale and fibrous. There was extension to surrounding soft tissue (Fig. 3b).

**Fig. 4:**
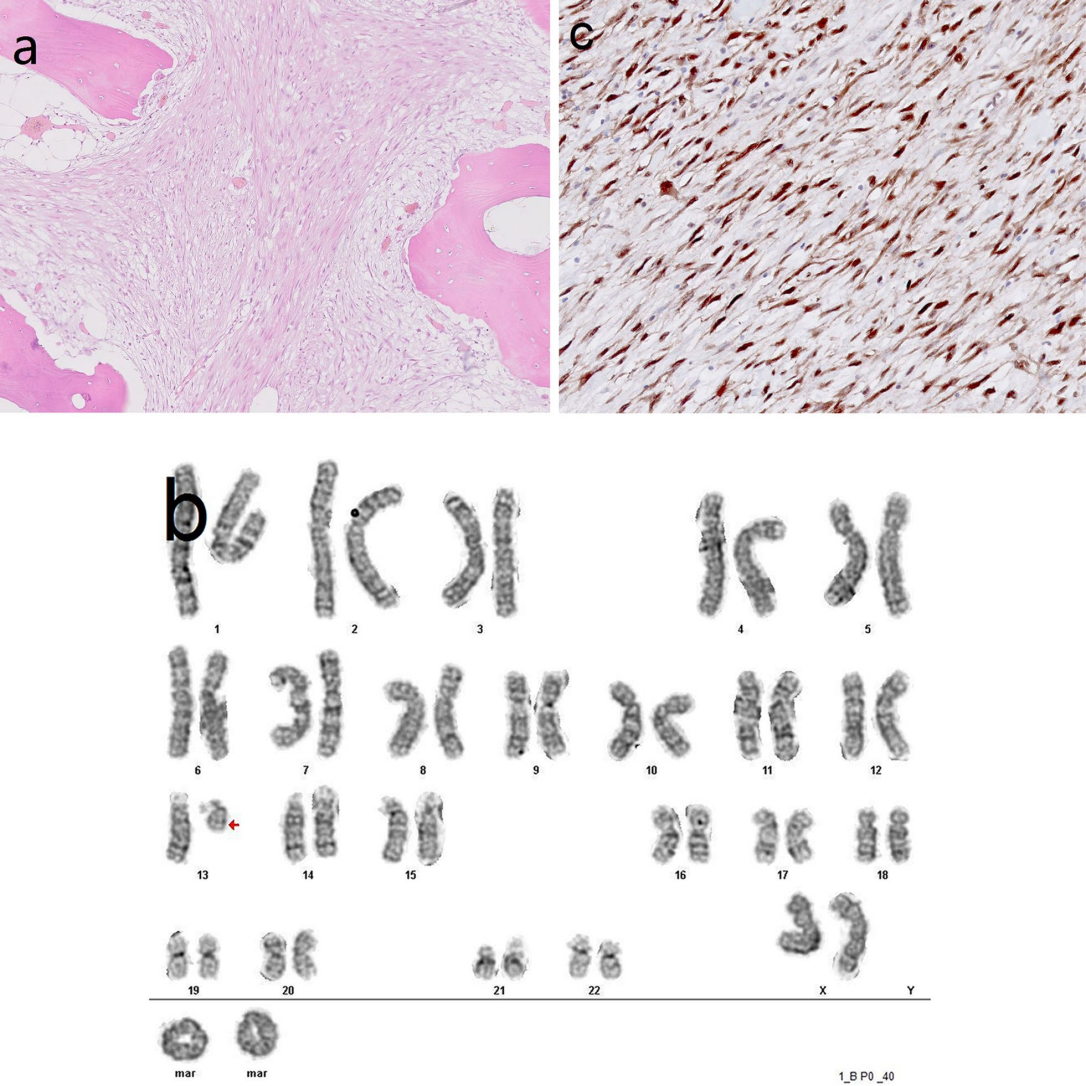
Histology, karyotype and CDK4 expression in DF-LGOS. **a** Histology showing a fibroblastic tumor with permeative invasive growth (H&E, original magnification × 100). As such, the histology of DF-LGCOS resembles that of DF, shown in Fig. 2a. **b** DF-LGOS karyotype: 47~49,XX,del(13)(q12q32),+1~2r,+1~2mar,1dmin[cp17]/46,XX[2]. **c** Diffuse nuclear expression of CDK4 in cell nuclei of DF-LGCOS (original magnification × 200)

Tumor histology strongly resembled the desmoplastic fibroma diagnosed in case 1, however, with some differences. As shown in Fig. 4a, this tumor also consisted of bundles of moderate cellular tissue, with fibroblast-like, spindle cells in abundant collagenous stroma. However, there was evidence of invasive growth in trabecular bone and surrounding skeletal muscle tissue. Although nuclear chromatin was bland, few normal mitoses were found. Osteoid or trabecular bone was absent.

As depicted in Fig. 4b, cytogenetic analysis showed an abnormal karyotype: 47~49,XX,del(13) (q12q32),+ 1~2r,+1~2mar,1dmin [cp17]/46,XX [2]. This encompasses an interstitial deletion of the long arm of chromosome 13 (q12q32), consistent with heterozygous loss of the *RB1* tumor suppressor gene. With cancer hotspot NGS analysis we found amplification of *CDK4* (NM_000075.3) and an imbalance of the *RB1* gene on chromosome 13.

With IHC, tumor cells exhibited strong nuclear staining for CDK4 (Fig. 4c) and moderate nuclear staining for SATB2. RB1 expression was heterogeneous, not completely lost.

In this case a conclusive diagnosis of DF-LGOS could be made, based on histologic features (an invasive fibroblastic tumor with mitotic activity), karyotyping (heterozygous loss of *RB1*) and molecular genetics/IHC (*CDK4* amplification).

## Discussion and conclusions

We have presented the clinical presentation, imaging studies, gross and microscopic pathology, IHC, cytogenetics and molecular genetics (cancer hotspot analysis) of DF and DF-LCOS, two very rare bone tumors, which closely resemble each other.

DF is very rare indeed. Among 4692 benign bone tumors treated in the Birmingham Royal Orthopedic Hospital, Evans et al. [1] identified 13 cases of DF, an incidence of 0.003%. Böhm et al. [2] reviewed 189 cases of DF reported in the literature up to 1996 and observed that, although DF occurs at all ages, children and young adults are most commonly affected, three-quarter of patients being younger than 31 years. Sex distribution is almost equal. DF most commonly presents in the mandible (22%), but also in pelvic bones (13%), and long bones—femur (15%), radius (12%), and tibia (9%). Notably, pathologic fracture of a long bone was reported in 12% of patients. Thus, the clinical presentation of our DF case as a tumor in the distal radius of a 10-year-old girl, who had experienced a radius fracture 3 years earlier, matches data from the literature.

It is well appreciated that DF has a high recurrence rate after intralesional excision [1, 2], but since DF is a benign tumor that does not metastasize, we choose to remove the radius tumor of this young girl intralesionally, in order to preserve arm and wrist function. Unfortunately, a recurrence has occurred 12 months after surgery.

As our case illustrates, DF may present as a slowly progressive but locally aggressive tumor. As reviewed by Nedopil et al. [3] by imaging studies, DF can show cortical breakthrough and extension in surrounding soft tissue.

Moreover, infiltrative tumor growth may be seen by microscopy. Mitoses are only rarely found, an important criterion to discriminate DF from DF-LCOS or low-grade fibrosarcoma [4].

Cytogenetic analysis of our DF case revealed a normal female karyotype in 18 cells, with a trisomy 8 detected in 2 cells. To our knowledge, only two papers have been published on the cytogenetics and molecular genetics of DF. Bridge et al. [5] found trisomies 8 and 20 in a single case of DF, but again, these cytogenetic abnormalities were also detected in other fibro-osseous bone tumors, by which these are noncontributory to a certain DF diagnosis.

An abnormal karyotype 46,XX,del(11)(q13q23),der(19)t(11;19)(q13;p13)del(11)(q23) was reported by Trombetta et al. [6] in a DF occurring in the femur of a 20-year-old female patient. It was hypothesized that loss of a genomic region in 11q, an area containing the genes *RBM14, RBM4, RBM4B, SPTBN2,* and *C11orf80* may be of pathogenic significance.

Using IHC, others and we have noticed nuclear staining of beta-catenin in DF [3, 4, 7–11]. However, although nuclear expression of beta-catenin supports a diagnosis of DF, one has to be aware that nuclear immunostaining of beta-catenin also occurs in other fibro-osseous bone tumors [9] or fibrous soft tissue tumors [12]. Moreover, IHC for beta-catenin is not specific for *APC/CTNNB1* mutations in fibro-osseous bone tumors. *CTNNB1* mutations are a rare molecular event in the few cases of DF that have been analyzed [7–9]. In fact, Flucke et al. [8] found a p.T41A *CTNNB1* mutation in 1 out of 2 cases of DF arising in the mandible, Horvai and Jordan [9] found an *APC* mutation, but no *CTNNB1* mutation in a single DF analyzed, and Hauben et al. [7] found no *CTNNB1* mutation in six DF cases. Using NGS, we detected a *CTNNB1* hotspot class 5 pathogenic variant in exon 3: p.S45F, which is a gain of function mutation. Clearly, to be able to estimate the real frequency of *CTNNB1* mutations in DF, more cases have to be studied, preferably using NGS, since NGS has a higher sensitivity compared with traditional DNA sequencing methods in picking up *CTNNB1* mutations [13] Interestingly, the S45F *CTNNB* mutation also occurs in desmoid fibromatosis, in particular in aggressive and recurrent lesions [14]. However, it remains to be proven that DF is the bony counterpart of desmoid fibromatosis of soft tissue. In this respect, one may argue whether our case 1 represents a soft tissue tumor that had invaded bone. However, given the imaging features, in particular the bubbly compartmentalized appearance of the radius tumor and the bowing of the distal ulna without bone invasion (see Fig. 1), we regarded the radius tumor in this girl as a slow growing primary bone tumor with soft tissue extension, a clinical presentation consistent with a histopathologic diagnosis of desmoplastic fibroma of bone.

The majority of central osteosarcomas are high-grade conventional osteosarcomas, in which the tumor cells show severe nuclear atypia and produce a variable amount of cartilaginous or osteoid matrix. High grade osteosarcomas with severe nuclear atypia, but little or no matrix formation can be confirmed by SATB2 immunohistochemistry [15, 16].

Our DF-LGCOS case is part of another subset of low grade central osteosarcomas namely the ones that resemble DF and have little or no osteoid matrix deposition. So far, this very rare OS subtype has only been described in case reports and small series [17, 18]. Most likely these rare DF-LCOS have been included in the histological spectrum of fibrosarcomas of bone [4]. We agree with Horvai and Jordan [9], who stated that it seems logical that at least a subset of fibrosarcomas of bone are actually osteosarcomas with little or no osteoid or bone formation. Surprisingly, in the 2013 WHO classification of tumors of soft tissue and bone, fibrosarcomas of bone are defined as intermediate to high grade spindle cell tumors that lack any line of differentiation other than fibroblastic, leaving little room for the recognition of low grade variants, also excluding DF- LGCOS.

Strong and diffuse SATB2 nuclear IHC staining reflects an osteoblastic line of differentiation. To date, only one Chinese study investigated SATB2 expression in low grade osteosarcoma and desmoplastic fibroma. These authors found that low-grade osteosarcoma and fibrous dysplasia are positive for SATB2, while desmoplastic fibroma, low-grade fibrosarcoma and other fibrous tumors are negative [19].

The notion that DF-LGCOS is an osteosarcoma variant is also supported by the cytogenetics and molecular genetics of our second case. DF-LGCOS had a karyotype with interstitial deletion of the long arm of chromosome 13 (q12q32), consistent with loss of the *RB1* tumor suppressor gene, a genetic abnormality found in a substantial number of osteosarcomas. Notably, cancer hotspot NGS analysis revealed amplification of *CDK4* and IHC showed overexpression of CDK4.

The prototypical LGCOS (which resembles parosteal osteosarcoma) usually produces abundant bone matrix and contains trabecular woven bone. The fibroblastic stromal cells of the prototypical LGCOS show slight nuclear atypia and mitosis are not easily discerned. This subset of LGCOS often has gain or amplification of the *MDM2* and/or *CDK4* genes, which can be visualized by their nuclear expression by IHC [19]. By IHC, CDK4 is positive in the majority of LGOS, and, when combined with MDM2 immunostaining, the sensitivity and specificity for LGOS is 100% and 97.5%, respectively [20].

The clinical presentation, imaging studies and gross morphology of DF-LGCOS shows much overlap with DF. Both are fibrous tumors of bone with are slowly progressive and locally aggressive showing cortical breakthrough. DF and DF-LGCOS consist of bundles of moderately cellular collagenous tumor tissue with spindled fibroblast-like cells. The two cases reported herein show that karyotyping and molecular genetic analysis may contribute to a conclusive diagnosis, DF showing *CTNNB1* S45F mutation and DF-LGCOS showing *CDK4* amplification.

## Abbreviations

DF: desmoplastic fibroma
DF-like LGCOS: low-grade central osteosarcoma (LGCOS) resembling desmoplastic fibroma
NGS: next generation sequencing
IHC: immunohistochemistry
LGCOS: low-grade central osteosarcoma
CC1: cell conditioning buffer 1
